# Beginner's Guide on the Use of PAML to Detect Positive Selection

**DOI:** 10.1093/molbev/msad041

**Published:** 2023-04-25

**Authors:** Sandra Álvarez-Carretero, Paschalia Kapli, Ziheng Yang

**Affiliations:** Department of Genetics, Evolution and Environment, University College London, London, United Kingdom; Department of Genetics, Evolution and Environment, University College London, London, United Kingdom; Department of Genetics, Evolution and Environment, University College London, London, United Kingdom

**Keywords:** adaptive evolution, *d*_N_/*d*_S_, nonsynonymous substitutions, PAML, positive selection, synonymous substitutions

## Abstract

The CODEML program in the PAML package has been widely used to analyze protein-coding gene sequences to estimate the synonymous and nonsynonymous rates (*d*_S_ and *d*_N_) and to detect positive Darwinian selection driving protein evolution. For users not familiar with molecular evolutionary analysis, the program is known to have a steep learning curve. Here, we provide a step-by-step protocol to illustrate the commonly used tests available in the program, including the branch models, the site models, and the branch-site models, which can be used to detect positive selection driving adaptive protein evolution affecting particular lineages of the species phylogeny, affecting a subset of amino acid residues in the protein, and affecting a subset of sites along prespecified lineages, respectively. A data set of the myxovirus (*Mx*) genes from ten mammal and two bird species is used as an example. We discuss a new feature in CODEML that allows users to perform positive selection tests for multiple genes for the same set of taxa, as is common in modern genome-sequencing projects. The PAML package is distributed at https://github.com/abacus-gene/paml under the GNU license, with support provided at its discussion site (https://groups.google.com/g/pamlsoftware). Data files used in this protocol are available at https://github.com/abacus-gene/paml-tutorial.

## Introduction

The neutral theory of molecular evolution suggests that most of the observed variation within and between species is not due to natural selection, but rather to random fixation of mutations with little fitness significance (Kimura 1968; King and Jukes 1969). In other words, advantageous mutations are rare at the molecular level. However, advantageous mutations in genes and genomes are ultimately responsible for shaping the morphology, behavior, and physiology of the species, or for species divergences and evolutionary innovations. Detecting molecular adaptation thus allows us to achieve a better understanding of the evolutionary process. Studying molecular adaptation is currently more exciting than ever given the availability of vast genomic data and computational resources, as it is now possible to systematically interrogate the genomes for signatures of positive selection across a wide range of organisms.

In protein-coding genes, we can distinguish the *synonymous* or *silent* substitutions (nucleotide substitutions that do not change the encoded amino acid) from the *nonsynonymous* or *replacement* substitutions (those that do change the amino acid). Because natural selection operates mainly at the protein level, synonymous and nonsynonymous mutations are under very different selective pressures and fixed at very different rates. Thus, with the synonymous rate acting as a reference point, one can tell whether fixation of nonsynonymous mutations in the population is accelerated or decelerated by natural selection acting on the protein. Comparison of synonymous and nonsynonymous substitution rates can therefore reveal the direction and strength of natural selection acting on the protein (Kimura 1977; Miyata and Yasunaga 1980). A gene with accelerated nonsynonymous substitution rate, indicated by the nonsynonymous/synonymous rate ratio *d*_N_/*d*_S_ > 1, is said to be under positive selection. This kind of test is in particular effective in detecting diversifying selection or balanced selection, as it uses excessive nonsynonymous substitutions as evidence that natural selection has aided the fixation of nonsynonymous mutations. Tests based on *d*_N_/*d*_S_ may be less effective when applied to data from the same species because of lack of sequence divergences and because of complications in the interpretation of the *d*_N_/*d*_S_ ratio (Kryazhimskiy and Plotkin 2008).

With protein-coding gene sequences available from different species, one may be able to ask the following questions. Are there any codons that are evolving under positive selection with *d*_N_/*d*_S_ > 1 and, if there are, how can we identify them? Is there positive selection driving fast replacement codon substitution along certain lineages, for example, right after gene duplication or when a species became adapted to a new environment? What kind of statistical tests are suitable for answering such questions, and how does one estimate the strength of selection?

A number of Markov chain models have been developed for detecting positive selection affecting genes, amino acid residues, or evolutionary lineages (Kosakovsky Pond et al. 2005; Yang 2007). In this protocol, we discuss the use of codon models in maximum-likelihood phylogenetic analyses of sequence alignments to detect positive selection driving the fixation of advantageous nonsynonymous mutations.

## Detection of Adaptive Molecular Evolution Under Models of Codon Substitution

Several codon-substitution models have been implemented in the software CODEML, part of the PAML package (Yang 2007), as extensions of the Goldman and Yang (1994) and Muse and Gaut (1994) models. The main feature of codon-substitution models, compared with models of nucleotide or amino acid substitution, is that the codon triplet is considered the unit of evolution (Goldman and Yang 1994). The commonly used version of the model (e.g., Yang 1998; Yang and Nielsen 1998) ignores chemical differences between amino acids and uses the same nonsynonymous/synonymous rate ratio (i.e., *ω* = *d*_N_/*d*_S_) that does not depend on the source and target amino acids encoded by the codons. This assumption simplifies the interpretation of the model considerably. Specifically, the *ω* ratio measures the direction and magnitude of selection on amino acid changes: values of *ω* < 1, = 1, > 1 indicate negative purifying selection, neutral evolution, and positive selection, respectively. However, the *ω* ratio averaged over all sites of a gene and across all lineages (branches) on the phylogeny is seldom greater than 1, and therefore, its use to detect positive selection has virtually no power. Instead, detecting positive selection that affects specific branches or individual sites has proven more useful.

*Branch* models assume different *ω* ratio parameters for different branches on the phylogeny (Yang 1998; Yang and Nielsen 1998). They may be used to detect positive selection acting on particular lineages, without averaging the *ω* ratio throughout the phylogenetic tree. For instance, they are useful for detecting positive selection after gene duplications, where one copy of the duplicates may have acquired a new function, and thus may have evolved at an accelerated rate.

*Site* models treat the *ω* ratio for any site (codon) in the gene as a random variable from a statistical distribution, thus allowing *ω* to vary among codons (Nielsen and Yang 1998; Yang et al. 2000). Positive selection is defined as the presence of some codons at which *ω* > 1. A likelihood ratio test (LRT) is performed to compare a null model that does not allow for any codons with *ω* > 1 against a more general model that does. Two pairs of site models are particularly effective, which form two widely used tests for positive selection using CODEML (table 1): (1) **M1a** (*Nearly Neutral*) vs. **M2a** (*Positive Selection*) (Nielsen and Yang 1998; Wong et al. 2004; Yang et al. 2005) and (2) **M7** (beta) vs. **M8** (beta*&ω*) (Yang et al. 2000). The LRT statistic, or twice the log-likelihood difference between the two compared models (2Δℓ), may be used in a chi-square test test with the degree of freedom to be the difference in the number of free parameters between the two models. For example, **M1a** has 2 free parameters and **M2a** has 4, so the degree of freedom is 2, and the critical values for the chi-square test are $\chi_{2,5\text{\%}}^{2}=5.99$ at 5% significance level and $\chi_{2,1\text{\%}}^{2}=9.21$ at 1% significance level. The **M7**-**M8** comparison also has 2 degrees of freedom (table 1).

**Table 1.** Site Models for Testing Positive Selection Affecting Amino Acid Residues in a Protein.

Site model	Free parameters^a^	Number of site classes	Model parameters	Model comparison (LRT)
**M1a**	2 (*p*_0_, *ω*_0_)	2	*p*_0_ (*p*_1_ = 1 − *p*_0_), *ω*_0_ < 1, *ω*_1_ = 1	**M1a** vs. **M2a**: df = 2
**M2a**	4 (*p*_0_, *p*_1_, *ω*_0_, *ω*_2_)	3	*p*_0_, *p*_1_ (*p*_2_ = 1 − *p*_0_ − *p*_1_), *ω*_0_ < 1, *ω*_1_ = 1, *ω*_2_ > 1	
**M7**	2 (*p*, *q*)	10	beta(*p*, *q*)	**M7** vs. **M8**: df = 2
**M8**	4 (*p*_0_, *p*, *q*, *ω*_*s*_)	11	*p*_0_, (*p*_1_ = 1 − *p*_0_), beta(*p*, *q*), *ω*_*s*_ > 1	

a Number of free parameters in the *ω* distribution. **M1a**: *p*_0_ is the proportion of sites with *ω*_0_ < 1, while *p*_1_ =1 − *p*_0_ is the proportion of sites with *ω*_1_ = 1. **M2a**: same as **M1a** but includes an additional class of sites with *ω*_2_ > 1 in proportion *p*_2_, with *p*_0_ + *p*_1_ + *p*_2_ = 1. **M7**: the model uses a beta distribution with parameters *p* and *q* to describe variable *ω* for sites in the range 0 ≤ *ω* ≤ 1. **M8**: *p*_0_ is the proportion of sites with *ω* from beta(*p*, *q*) as in **M7**, but an additional class is now added (with proportion *p*_1_) with *ω*_*s*_ > 1.

If the LRT favors **M2a** or **M8**, we may ask: which sites in the gene are under positive selection? This question is answered by using the Bayes theorem to calculate the posterior probability that each site is from the class of positive selection with *ω* > 1. Two approaches are implemented in CODEML. The Naïve Empirical Bayes (NEB) method (Nielsen and Yang 1998) calculates the posterior probabilities that each site is from the different site classes by using the maximum-likelihood estimates (MLEs) of parameters in the model without accounting for their sampling errors or uncertainties. The Bayes empirical Bayes (BEB) method is an improvement over NEB and accommodates the uncertainties in the MLEs (Yang et al. 2005). While CODEML reports results from both methods, the BEB should be used instead of NEB.

*Branch-site* models aim to detect positive selection that affects only a few sites on prespecified lineages (Yang and Nielsen 2002). Branches under test for positive selection are called *foreground* branches, whereas all other branches on the tree are the *background* branches. For the background branches, there are two classes of sites, the conserved sites with 0 < *ω*_0_ < 1 and the neutral sites with *ω*_1_ = 1. For the foreground branches, some of those sites become under positive selection with *ω*_2_ > 1. Positive selection or the presence of sites with *ω*_2_ > 1 is tested by comparing this model with a null model in which *ω*_2_ = 1 is fixed, using a 50:50 mixture of 0 and $\chi_{1}^{2}$ as the null distribution (Yang et al. 2000; Zhang et al. 2005). As with site models, the BEB method can be used to identify codon sites potentially under positive selection on the foreground branches.

This branch-site model in common use is the modified version of the old branch-site models A and B of Yang and Nielsen (2002). The old model A fixes *ω*_0_ = 0 for the conserved sites and *ω*_1_ = 1 for the neutral sites, which is unrealistic as it does not accommodate sites under constraints with 0 < *ω* < 1. The old model B uses both *ω*_0_ and *ω*_1_ as free parameters and may have high false positives when the estimated *ω*_1_ is slightly greater than 1 and when sites under weak constraints are assigned to such a class of positive selection. The modified branch-site model A deals with both issues by having 0 < *ω*_0_ < 1 as a free parameter for conserved sites, and by fixing *ω*_1_ = 1 to account for sites that are nearly neutral or under weak constraint (Yang et al. 2000, 2005; Zhang et al. 2005).

It is important to note that foreground branches should be specified *a priori*. If multiple branches on the tree are tested for positive selection when using the same dataset without *a priori* biological hypothesis, a correction for multiple testing may be required (Anisimova and Yang 2007). The Bonferroni correction may be too conservative, and the Rom's procedure (Rom 1990) has a slightly higher power and is preferred (Anisimova and Yang 2007). One may also use procedures that control the false discovery rate (FDR), which is the expected proportion of true nulls among all rejected null hypotheses or the proportion of false positive results among all positive test results (Benjamini and Hochberg 1995, 2000). Note that, if sequences are extremely divergent or there are serious model violations, multiple testing correction may be unreliable (Anisimova and Yang 2007).

In this protocol, we provide step-by-step guidelines for performing analyses under the aforementioned models. In particular, we focus on conducting four types of analyses using CODEML: (1) calculation of *ω* as a measure of average selective pressure on the gene under the homogenous model of one *ω* for all sites and branches, (2) detecting positive selection affecting a subset of sites in the coding sequence evolving under positive selection (*site* model), (3) detecting a specific branch or branches of a phylogeny evolving under positive selection (*branch* model), and (4) detecting a subset of sites for particular branches (*branch-site* model). We illustrate the analyses using an alignment of myxovirus sequences for mammals and birds (Hou et al. 2007). Last, we discuss the latest implementation in CODEML, which enables tests for positive selection with genomic data sets consisting of thousands of gene alignments.

## Protocol

### Obtaining and Compiling the PAML Suite of Programs

All the analyses of the protocol are carried out using the latest version of CODEML, part of the PAML package (PAML v4.10.6 at the time of writing), which can be downloaded from the PAML GitHub repository (https://github.com/abacus-gene/paml). Instructions for installing PAML on different operating systems can be found at http://abacus.gene.ucl.ac.uk/software/.

Throughout the protocol, we show how to execute CODEML from the command line in a UNIX (e.g., Mac OSX) and Linux environment (e.g., Ubuntu, Centos, etc.). Windows users may run CODEML from the Windows Command Prompt or from an Ubuntu terminal enabled in the Windows Subsystem for Linux (WSL), available on Windows 10 or later. Short tutorials for using the terminals are available at the following links:

http://abacus.gene.ucl.ac.uk/software/CommandLine.Windows.pdf

http://abacus.gene.ucl.ac.uk/software/CommandLine.MACosx.pdf

We assume that the path to the executable file has been correctly exported to the users’ system (i.e., added to the PATH global variable, which can be usually defined through the ∼/.bashrc or ∼/.bash_profile hidden files), and therefore CODEML can be executed just by typing the name of the executable file on the terminal (i.e., codeml). If that is not the case, the full path to the executable file should be provided.

### Running CODEML

**Fig. 1.**
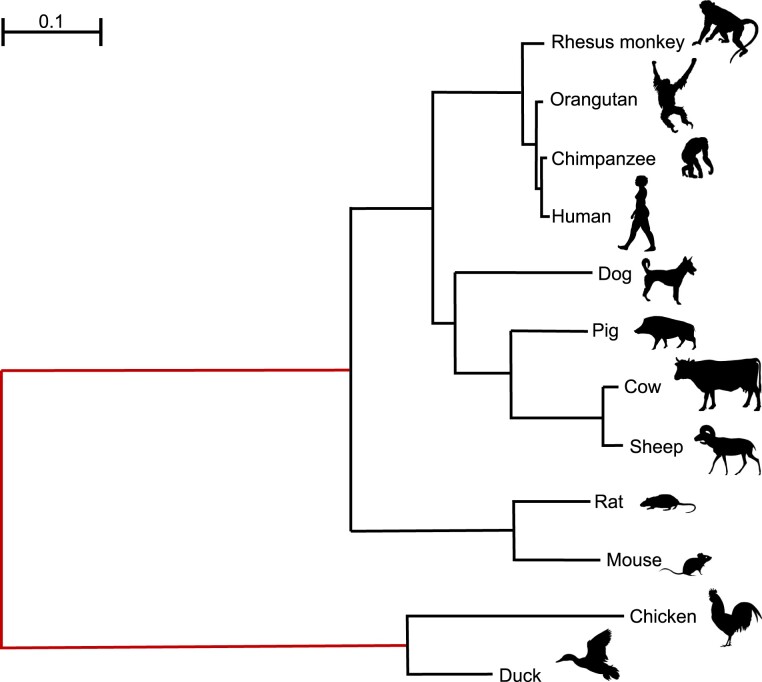
Phylogenetic tree for ten mammals and two bird species reconstructed by maximum-likelihood (ML) under the GTR + G model with RAxML v8.2.10 using the *Mx* gene sequences. The best-scoring ML tree is unrooted, but the root is shown for clarity. The chicken and duck branches were identified as the foreground branches in the branch and branch-site tests of positive selection. When the model assumes the same evolutionary process for the two branches around the root (e.g., if both are assumed to be the background branches in the branch or branch-site models), the root of the tree will be identifiable. Then, the two branches should be merged into one (branches shown in red), with one branch length estimated. In other words, the unrooted tree should be used. However, if the two branches are assumed to evolve differently in the model (e.g., if one branch is a background branch and the other is labeled as foreground in the branch or branch-site models), the root of the tree is then identifiable, and the rooted tree should be used. All silhouettes are from https://www.phylopic.org/.

For all the analyses presented in this protocol, we use a dataset of 12 sequences of the myxovirus (*Mx*) gene for ten mammal and two bird species, generated and analyzed previously by Hou et al. (2007) (see fig. 1). The *Mx* gene is involved in the antiviral response in the host species. Hou et al. (2007) hypothesized that the gene had evolved under positive selection in the chicken and duck lineages, while it was under strong purifying selection in the mammal lineages.

We have created a GitHub repository (https://github.com/abacus-gene/paml-tutorial/tree/main/positive-selection) as Supplementary material where we provide a step-by-step tutorial detailing how to download and filter the myxovirus sequence data, and then generate the gene alignment that we use throughout this protocol.

### The Control File

**Fig. 2.**
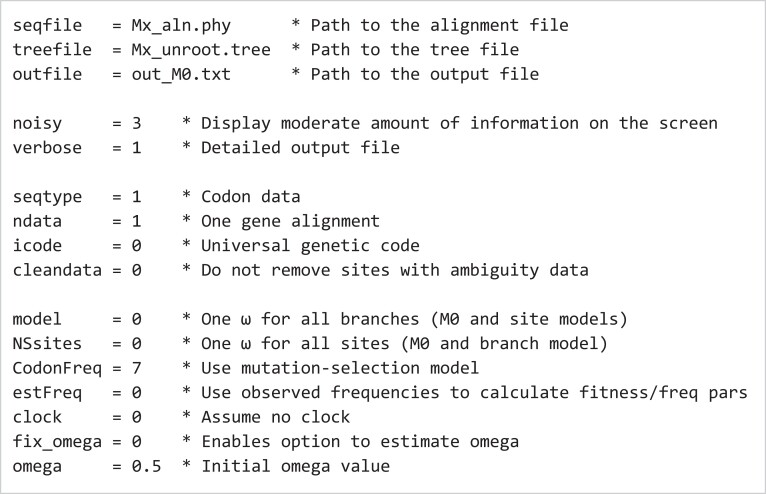
Example CODEML control file. The first three blocks specify the paths to the input and output files (lines 1–3), how much information is to be printed on the screen or in the output file (lines 5–6), and the data type of the sequence alignment (lines 8–11). The fourth block defines the evolutionary model. In this example, a homogeneous *ω* across both branches and sites is selected (model=0, NSsites=0). Several models are available for accounting for unequal codon usage with CodonFreq = 0 (**Fequal**), 1 (**F1 × 4**), 2 (**F3 × 4**), or 3 (**Fcodon**). Here, we use the mutation-selection model with observed codon frequencies used as estimates (CodonFreq=7, estFreq=0) (Yang and Nielsen 2008). This model explicitly accounts for the mutational bias and selection affecting codon usage, and is preferable over the other models concerning codon usage (Yang and Nielsen 2008). We estimate *ω* from the data and so choose fix_omega=0, with the initial value omega=0.5. Lastly, the evolutionary rate is allowed to vary among lineages on the tree (i.e., clock=0).

The control file contains the information required for CODEML to run any analysis. A detailed description of the variables in the control file is provided in the PAML documentation. Here, we focus on the variables that are relevant to the analyses detailed in this protocol (see fig. 2).

We note a few rules about the control file:

1. The control file is read line by line. If the same variable is specified twice, the second one overwrites the first.
2. Some specifications in the control file are common to all substitution models.
3. Blank lines are ignored by the program. Anything on the same line after an asterisk (*) is treated as comments.

#### The Input/Output Files

The first block of the example control file (see fig. 2) specifies the paths to the input files, those with the sequence alignment (seqfile) and the phylogenetic tree (treefile), and the output file in which the main results of the analysis are to be saved (outfile). The alignment file should be in the PHYLIP format. The header (line that precedes the alignment) details the number of taxa and the length of the alignment. For instance, the file with the *Mx* gene alignment used in this protocol has 12 species and 1,989 base pairs, so the header is “12 1989”. If more than one gene alignment is included in the input file, each gene alignment will be assumed to start after a header line. The tree file contains the tree topology in Newick format (bootstrap values should be deleted if present) and may include a header. For instance, for a Newick tree of 12 taxa, the header “12 1” should be added before the Newick tree. Branch lengths are not required and, given that they might interfere with additional information (e.g., node labels) in some models, we suggest they are not included in the Newick tree. If more than one tree is listed in the tree file, a header is necessary. For a discussion on the data format, see section “*Alignment, sequence data file, and tree*” in the Supplementary material and our GitHub repository (https://github.com/abacus-gene/paml-tutorial/tree/main/positive-selection/00_data).

#### Screen Output

The amount of information to be printed out on the screen or in the output file is controlled by the variables noisy and verbose.

#### Input Sequence Data

The type of data to be used in the inference (e.g., nucleotides, amino acids, or codons that are to be translated into amino acids by CODEML) is specified with the variable seqtype. The number of genes or alignments are defined with the variable ndata, whereas the genetic code is specified with the variable icode. In addition, the variable cleandata can be used to decide if sites with ambiguity data and alignment gaps should be kept (cleandata=0) or removed (cleandata=1). Prior to using CODEML, sequences must be properly aligned, with introns, noncoding regions, and stop codons removed. To preserve the reading frame, a useful strategy is to first align protein sequences and then construct the codon alignment accordingly. We recommend removing alignment columns or regions that are predominantly gaps or are hard to align, and then use cleandata=0 to preserve information in the data. See also recommendations in sections “*Alignment, sequence data file, and tree file*” and “*Gene tree versus species tree*” in the Supplementary material.

#### Substitution Model

Several variables are used to specify the model to be used in the analysis. First, *ω* may vary among lineages (model), across sites (NSsites), or across both lineages and sites (model and NSsites). Second, the codon frequencies (CodonFreq) can be either equal (1/61 each) or different to account for codon usage bias. The FmutSel model (CodonFreq = 7) assigns a fitness to every codon with 60 (= 61–1) codon fitness parameters for the universal genetic code (Yang and Nielsen 2008). The FmutSel0 model (CodonFreq=6) is a special case of FmutSel and assigns the same fitness value for synonymous codons, so that only 19 (= 20–1) amino acid fitness parameters are used. The model assumes that amino acid frequencies are determined by the functional requirements of the protein, but the relative frequencies of synonymous codons are determined solely by the mutational-bias parameters. Under those mutation-selection models, the variable estFreq specifies the use of observed codon frequencies from the data or estimation by maximum likelihood. Depending on the model defined with those parameters, the value of *ω* can be fixed or estimated through the variables fix_omega and omega. The variable clock is used to enforce the molecular clock (i.e., rate constancy among lineages). As the models we discuss here are all time-reversible and as we do not assume the molecular clock (clock=0), an unrooted tree should be used. Note that virtually all phylogenetic programs including RAxML (Stamatakis 2014; Kozlov et al. 2019), IQ-TREE (Minh et al. 2020), and MrBayes (Ronquist et al. 2012) produce unrooted trees. The strict-clock and local-clock models are implemented in CODEML by using clock=1 and clock=2, but these are not used in this protocol. For a discussion of whether rooting or not the tree, see section “*Rooted tree versus unrooted tree*” in the Supplementary material.

### One Ratio With Homogeneous ω Across Lineages and Sites

**Fig. 3.**
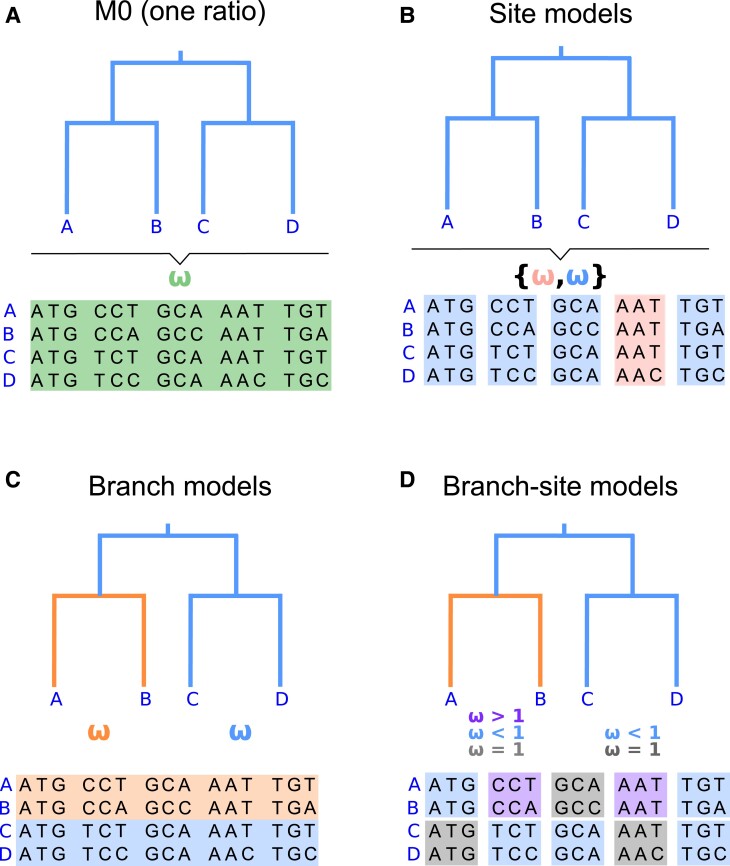
Illustration of four different types of models implemented in CODEML. (*A*) homogeneous evolutionary pressure throughout the history of the gene (**M0**: one ratio, with one *ω* ratio for all sites and branches, specified as model=0 and NSsites=0); (*B*) heterogeneous pressure across codons (*site* models: model=0 and NSsites=1, 2, 7, 8, etc.); (*C*) heterogeneous pressure across branches of a tree but homogeneous across codons (*branch* model: model=2 and NSsites=0); and (*D*) heterogeneous pressure across sites and branches (*branch-site* model: model=2 and NSsites=2). See table 2 for more details on CODEML specifications.

The simplest codon model implemented in CODEML is **M0** (one-ratio), which assumes one *ω* ratio for all sites and across all lineages (fig. 3*A*). In most cases, this assumption is unrealistic, given that the majority of sites are expected to be under constraints with *ω* < 1. Thus, trying to identify evidence of positive selection under the **M0** model would fail. In fact, when our interest is to detect positive selection, the best practice is to estimate *ω* under the *site* or the *branch-site* models described later in the protocol. The **M0** model, however, gives us a null hypothesis that can be used as a reference for comparison to decide whether more complex models fit the data substantially better. The *ω* estimate under **M0** also gives an overall measure of selective constrain averaged over sites and lineages.

**Table 2.** Major Models Discussed in the Protocol and the Specifications in CODEML.

Model	CODEML specifications
**M0** (one ω for all sites and branches)	model = 0, NSsites=0
Branch model	model = 2, NSsites=0
Site models^a^	model = 0, NSsites=1 2 7 8
Branch-site model A	model = 2, NSsites=2

a The CODEML specifications detailed here will launch a batch run with four site models (M1a, M2a, M7, M8) that can be used to test for positive selection. To run site models individually, always set model=0 but change NSsites accordingly. For example **M1a**: NSsites=1; **M2a**: NSsites=2;**M7**: NSsites=7; **M8**: NSsites=8; etc.

To run CODEML using the control file codeml-M0.ctl of figure 2, we open a terminal, navigate to the folder containing the input files (the sequence alignment file, the tree file, and the control file), and then type:

codeml codeml-M0.ctl

The screen output should be self-explanatory, which includes information about the analysis, the alignment, and the progress of ML iterations. If there are errors in the input files or if the program fails for some other reasons, there may be error messages printed on the screen too. When the analysis is finished, some information about the data and the results will also be printed in the output text file (out_M0.txt). This output file is divided into five sections as explained below.

### Summary of the Site Patterns in the Input Alignment

The input alignment and its compressed version are printed at the top of the output file, where each site pattern is represented only once, and the corresponding frequencies (pattern counts) are shown in the block below. For example, in the gene alignment file used here (Mx_aln.phy), most site patterns are unique with frequency 1, whereas two site patterns are repeated two and four times as shown below. If cleandata=1 was used, both alignments *before* and *after* deleting the gaps and ambiguous sites would be printed, followed by the site pattern counts for the trimmed alignment. Note that if columns with gaps are removed, sites are renumbered, which may affect the output under the *site* or *branch-site* models.

**Figure:**
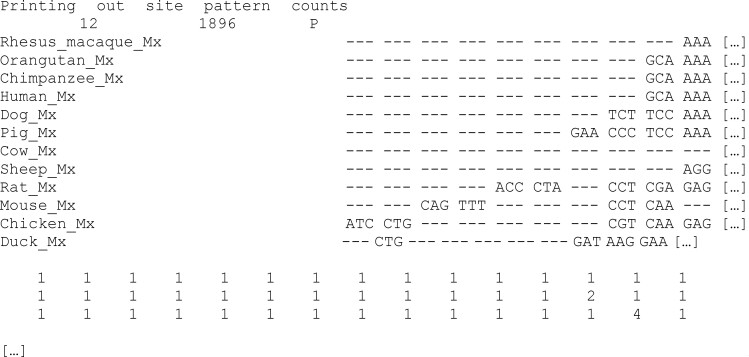


### Summary of the Input Alignment and the Model Selected

Next, the version of CODEML being used for the analysis is printed out together with the name of the alignment file. This is followed by the details about the model specified in the control file and the number of species (ns=12) and codons (ls=663, number of base pairs [1,989] divided into 3) in the alignment file:

CODONML (in paml version 4.10.6, November 2022) Mx_aln.phy

Model: One dN/dS ratio,

Codon frequency model: FMutSel

ns = 12 ls = 663

### Summary of Nucleotide and Codon Frequencies

Next, the observed nucleotide and codon frequencies for each sequence and their average over all sequences are printed in the output file, followed by the codon frequencies under the model, which could be used for simulation purposes using software such evolver (Yang 2007).

### Summary of Tree Scores and Estimated Model Parameter Values

The log-likelihood value, the number of free parameters, and the estimated branch lengths together with other parameters under the model (i.e., **M0** in this case) are shown in the first four lines below. Free parameters include branch lengths, the equilibrium frequencies, the transition/transversion rate ratio (*κ*), and the parameters in the omega distribution. Note that an unrooted tree with *n* tips has 2 × *n* − 3 branch lengths (i.e., 2 × 12 − 3 = 21 branch lengths in our tree of *n* = 12 taxa) and 3 mutation-bias parameters (four frequencies with the sum to be 1). It may be difficult to match the MLEs in line 4 to the corresponding branch lengths in the phylogeny or to the model parameters, but the branch lengths are printed again in the Newick tree. The last block shows the estimates of *κ* (transition/transversion rate ratio) and *ω*, as well as the mutation bias (nucleotide frequency) parameters. The mutation-selection model accommodates different codon frequencies by modeling mutational biases and fixations of mutations under selection: a higher mutation-bias parameter, say, $\pi_{A}^{*}$, means that the mutation process is biased toward A (Yang and Nielsen 2008, eqs. 1 and 4). Estimated values of *t* (branch lengths), *d*_N_ (nonsynonymous rate), and *d*_S_ (synonymous rate) for each branch follow. The output looks like the following:

**Figure:**
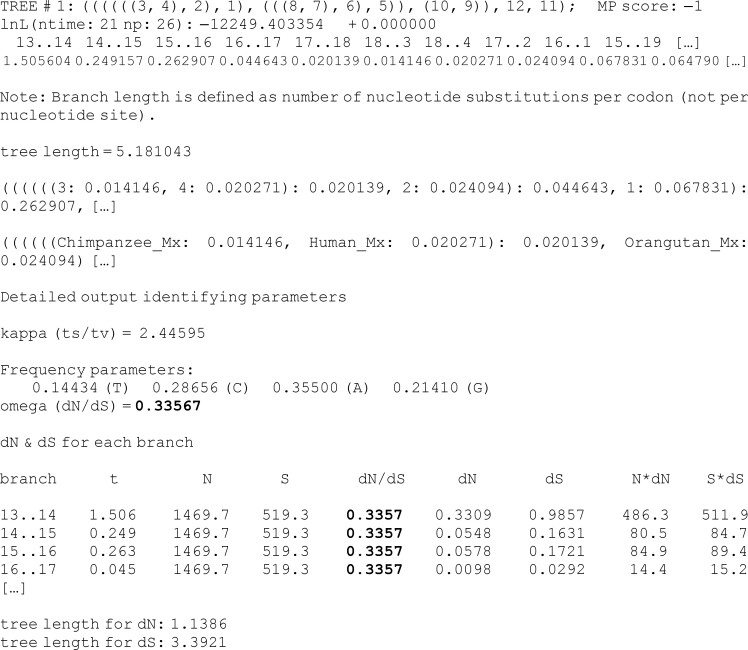


Since the homogenous model (**M0**) is specified (model = 0 and NSsites = 0), the same *ω* value (0.3357) is reported for each branch under the column dN/dS (see bold values above). The tree length is *d*_N_ = 1.1386 at the nonsynonymous sites and *d*_S_ = 3.3921 at the synonymous sites, with *ω* = *d*_N_/*d*_S_ = 0.3357, suggesting that the myxovirus gene is overall under purifying selection, with a nonsynonymous mutation having on average a third as large a chance (i.e., *ω* = *d*_N_/*d*_S_ = 0.3357) of going to fixation as a synonymous mutation.

In the next sections, we focus on three types of models useful for detecting positive selection: *site* models, *branch* models, and *branch-site* models.

### Site Models With Heterogeneous *ω* Across Sites

In this section, CODEML is run under various *site* models, which allow *ω* to vary across codons (fig. 3*B*). We can analyze the same data under several site models in a single run. In this example, we run CODEML under the homogeneous **M0** model (as before) and the following site-heterogeneous models: **M1a**, **M2a**, **M7**, and **M8** (see table 1). We edit the following control variables in the control file of figure 2 and rename it codeml-sites.ctl:

outfile = out_sites.txt * Path to the output file

NSsites = 0 1 2 7 8    * Models M0 (0), M1a (1), M2a (2), M7 (7), and M8 (8)

We can now execute CODEML from the command line:

codeml codeml-sites.ctl

The output file contains a section for each of the five models defined in the control file, where the log-likelihood values and the number of total parameters are provided, the latter being used to determine the degree of freedom for each model comparison.

To compare the nested site models, we use the LRT statistic, defined as twice the difference in log-likelihood between the null and alternative hypotheses, 2Δℓ = 2(ℓ_1_ − ℓ_0_), where ℓ_0_ is the log-likelihood score for the null model, whereas ℓ_1_ is the log-likelihood under the alternative model. The LRT statistic is compared with the *χ*^2^ distribution with the degree of freedom equal to the difference in the number of free parameters (which are given in the output file for each model) between the two models. The results are summarized in table 3.

**Table 3.** LRT of Positive Selection Under Site Models.

Model comparison	ℓ	Free parameters^a^	df	2Δℓ
**M0** vs. **M1a***(one-ratio vs. nearly neutral)*	ℓ_0_ = −12,249.40ℓ_1_ = −11,969.77	26 vs. 27	1	559.26
**M1a** vs. **M2a***(nearly neutral vs. positive selection)*	ℓ_0_ = −11,969.77 ℓ_1_ = −11,969.7	27 vs. 29	2	0
**M7** vs. **M8***(beta vs. beta&ω)*	ℓ_0_ = −11,944.13 ℓ_1_ = −11,937.85	27 vs. 29	2	12.54

Note.—The critical values are $\chi_{1,5\text{\%}}^{2}=3.84$ (i.e., 1 degree of freedom, df, at 5% significance), $\chi_{1,1\text{\%}}^{2}=6.63$ (i.e., 1 degree of freedom, df, at 1% significance), $\chi_{2,5\text{\%}}^{2}=5.99$ (i.e., 2 degree of freedom at 5% significance), and $\chi_{2,1\text{\%}}^{2}=9.21$ (i.e., 2 degree of freedom at 1% significance). The critical values can be calculated using the qchisq function in *R*. For instance, qchisq(p = 0.95, df = 1) returns $\chi_{1,5\text{\%}}^{2}=3.84$.

a Free parameters include branch lengths (21), the equilibrium frequencies (3), the transition/transversion rate ratio *κ* (1), and the parameters for the omega distribution. See table 1 for the free parameters in the *ω* distribution.

Models **M0** (one-ratio) and **M1a** (nearly neutral) are nested and can be compared using the LRT. This is a test for variability of selective pressure among amino acid sites rather than a test of positive selection. **M1a** fits the data much better than **M0**, with 2Δℓ = 559.26 $>\;\chi_{1,5\text{\%}}^{2}$, indicating that the selective pressure reflected by *ω* varies hugely among sites. Compared with **M1a**, **M2a** adds a class of sites under positive selection with *ω*_2_ > 1 (in proportion *p*_2_). This does not improve the fit of the model significantly as 2Δℓ = 0 (table 3). We performed an additional test for positive selection by comparing **M7** (beta, null model) against **M8** (beta&ω, alternative model). **M8** fits the data better than **M7** at the 5% significance level, with 2Δℓ = 12.54 $>\chi_{2,5\text{\%}}^{2}$ = 5.99, suggesting the presence of sites under positive selection with *ω* > 1. The test for sites under positive selection is thus equivocal with the **M1a**-**M2a** and **M7**-**M8** comparisons giving conflicting results. When the evidence for positive selection exists but is not very strong, the **M1a**-**M2a** test is noted to be more stringent, as sites under weak positive selection tend to be lumped into the site class with *ω*_1_ = 1 (Zhang et al. 2005).

The MLEs of parameters under the site models are in table 4. The parameter estimates can be found in the same sections of the output file that have been previously illustrated with the homogenous model. You may use bash scripts to extract those values from the output file. Alternatively, we include code snippets in the step-by-step tutorial in our GitHub repository (https://github.com/abacus-gene/paml-tutorial/tree/main/positive-selection/00_data).

**Table 4.** Log-likelihood Values and Parameter Estimates for the Site Models.

Model code	ℓ	*d*_N_/*d*_S_	Estimates of parameters^a^
**M0** (one-ratio)	ℓ = −12,249.40	0.336	*ω* = 0.336
**M1a** (nearly neutral)	ℓ = −11,969.77	0.501	*p*_0_ = 0.548 (*p*_1_ = 0.451) *ω*_0_ = 0.089
**M2a** (positive selection)	ℓ = −11,969.77	0.501	*p*_0_ = 0.548, *p*_1_ = 0.041 (*p*_2_ = 0.410), *ω*_0_ = 0.089
**M7** (beta)	ℓ = −11,944.13	0.391	*p* = 0.352, *q* = 0.548
**M8** (beta&ω)	ℓ = −11,937.85	0.436	*p*_0_ =0.942 (*p*_1_ = 0.058) *p* = 0.404, *q* = 0.750, *ω* = 1.841

a Parameters in parentheses are given by free parameters in the model (i.e., *p*_1_ = 1 − *p*_0_ and *p*_2_ = 1 − *p*_0_ − *p*_1_; see table 1).

Although **M0** assumes the same *ω* for all codons in the gene (i.e., model = 0 and NSsites = 0), the site models assume several site classes (fig. 3*A* and *B*). For example, under **M8** (beta&ω), 94.2% of sites (the estimated value of *p*_0_ = 0.942, see table 4 under **M8**) have *ω* from the beta (0.404, 0.750) distribution (the estimated values of the parameters of the beta distribution, *p* = 0.404 and *q* = 0.750, see table 4 under **M8**), whereas 5.8% sites (the estimated value of *p*_1_ = 0.058, see table 4 under **M8**) have *ω* = 1.841, which indicates the presence of a small proportion of amino acid residues under positive selection. This information can be found in the output file after the lines starting with the term MLEs. When multiple site models are used in a single run, each model will have its own block of output information where the parameter estimates are reported.

Under **M2a** and **M8**, the BEB method is used to calculate the posterior probability for each site coming from the different site classes. Sites with high posterior probabilities for the positively selected class are likely to be under positive selection. The output under **M8** looks like the following:

**Figure:**
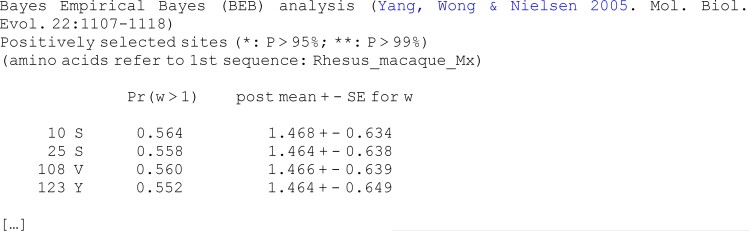


In each line, the first column shows the site position (e.g., 10, 25, 108, and 123), which is followed by the amino acid at this site in the first sequence (this is for identification of the site in the sequence). The third column (Pr (w > 1)) shows the posterior probability for the site to be from the positive-selection class (i.e., with *ω* > 1). The last columns show the posterior mean of *ω* and the standard deviation in the *ω* distribution for the site. Note that the BEB calculation is conducted under models of positive selection only (i.e., **M2a** and **M8**) but not under the null models (i.e., **M1a** or **M7**).

In our example, 14 sites had probability >50% for the positive-selection class with *ω* > 1, with 4 of them listed above. For instance, site 10 has amino acid serine in the first sequence, with probability 0.564 of coming from the positive-selection class, and the posterior distribution of *ω* for the site has mean 1.468 and SD 0.634. Here, the BEB calculation provided only weak evidence for sites under positive selection as the posterior probability for *ω* > 1 was low and the posterior distribution for *ω* was diffuse at every site. Also, the LRT is not significant for the **M1a-M2a** comparison and significant only at the 5% level under the **M7**-**M8** comparison. Together, these results suggest some evidence for sites under positive selection in the *Mx* gene, although this evidence is not very strong. See section “*BEB Analysis*” in the Supplementary material for an example where sites are positively selected.

### Branch Models With Heterogeneous *ω* Across Branches

In this section, we show how to run CODEML under *branch* models (fig. 3*C*), which assume that *ω* varies across the branches of the tree. The branch model is specified by labeling branches in the tree file using tags: #0 (default), #1, etc. The model currently allows a maximum of 8 branch types with different *ω* ratios. Here we consider only two types. The *foreground* branches are marked with the tag #1 (and assigned the ratio *ω*_1_) while all other branches are the *background* branches with the default tag #0 (and assigned the ratio *ω*_0_). Note that the default tag does not need to be included in the tree by the user. Users can either manually add the tags to locate the foreground branches in the Newick trees, use in-house scripts or code snippets such as the ones we provide in the GitHub repository (https://github.com/abacus-gene/paml-tutorial/tree/main/positive-selection/00_data), or use graphical tools such as PhyloTree (Shank et al. 2018) or EasyCodeML (Gao et al. 2019) to easily locate and label the foreground branches. Some tools might output a Newick tree with different tags to those used in CODEML, so users should check the tag format before running CODEML.

Here, we conduct four tests, designating different branches as the foreground: (1) chicken branch, (2) duck branch, (3) both chicken and duck branches, and (4) both chicken and duck branches as well as the branch ancestral to the chicken and duck in the rooted tree (i.e., subsequently referred to as the bird clade; table 5). The null model is **M0** in all tests, which assumes the same *ω* for all branches. The unrooted tree is used in all models except for the alternative hypothesis in test 4, in which the two branches around the root of the tree are assigned different *ω* ratios, and hence a rooted tree is needed. See section “*Rooted tree versus unrooted tree*” in the Supplementary material for details.

**Table 5.** Likelihood Ratio Test (LRT) Under Branch Models.

Model comparison	ℓ	Estimates of parameters	Free parameters^a^	df	2Δℓ
Test 1					
Null model (**M0**, unrooted tree)	ℓ_0_ = −12, 249.40	*ω* = 0.336	26	1	43.14
Alternative model (*branch* model, chicken as *foreground,* unrooted tree)	ℓ_1_ = −12, 227.83	*ω*_0_ = 0.293, *ω*_1_ = 1.171	27		
Test 2					
Null model (**M0**, unrooted tree)	ℓ_0_ = −12, 249.40	*ω* = 0.336	26	1	36.96
Alternative model (*branch* model, duck as *foreground,* unrooted tree)	ℓ_1_ = −12, 230.92	*ω*_0_ = 0.300, *ω*_1_ = 999	27		
Test 3					
Null model (**M0**, unrooted tree)	ℓ_0_ = −12, 249.40	*ω* = 0.336	26	1	51.03
Alternative model (*branch* model, chicken and duck simultaneously as *foreground,* unrooted tree)	ℓ_1_ = −12, 223.89	*ω*_0_ = 0.287, *ω*_1_ = 0.711	27		
Test 4					
Null model (**M0**, unrooted tree)	ℓ_0_ = −12, 249.40	*ω* = 0.336	26	2	17.15
Alternative model (*branch* model, bird clade as *foreground*, rooted tree)	ℓ_1_ = −12, 223.89	*ω*_0_ = 0.287, *ω*_1_ = 0.711	28		

Note.—The critical values are $\chi_{1,5\text{\%}}^{2}=3.84$ (i.e., 1 degree of freedom, df, at 5% significance), $\chi_{1,1\text{\%}}^{2}=6.63$ (i.e., 1 degree of freedom, df, at 1% significance), $\chi_{2,5\text{\%}}^{2}=5.99$ (i.e., 2 degree of freedom at 5% significance), and $\chi_{2,1\text{\%}}^{2}=9.21$ (i.e., 2 degree of freedom at 1% significance). The LRT statistic, 2Δℓ, is reported for all model comparisons.

a Free parameters include branch lengths (21 for an unrooted tree and 22 for a rooted tree), the equilibrium frequencies (3), the transition/transversion rate ratio *κ* (1); see section “Homogenous *ω* across sites and taxa” to learn how to calculate the number of free parameters. There is an extra parameter for **M0** (*ω*) and two additional ones for the branch model (one for the background branches,*ω*_0_, and another for the branch selected as foreground,*ω*_1_).CODEML has already been executed under the **M0** model, and hence we just need to use the results already generated for this analysis (please see section “Homogenous *ω* across sites and taxa*”* for a reminder).

In the first test, we assess whether the chicken lineage is under positive selection. The tree file is as follows, with the chicken branch labeled as foreground using the tag #1.

12 1

((((((Chimpanzee_Mx,Human_Mx),Orangutan_Mx),Rhesus_macaque_Mx),(((Sheep_Mx,

Cow_Mx),Pig_Mx),Dog_Mx)),(Mouse_Mx,Rat_Mx)),Duck_Mx,**Chicken_Mx #1**);

Then, we run the same analysis under the other three hypotheses. The control file for this analysis is similar as before, except for the following variables:

**Figure:**
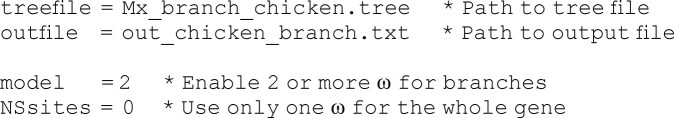


By setting model = 2, we specify that the foreground branches may evolve under a different *ω* value than the background branches. When running the other three analyses, please note that the tree and output file names should change accordingly. We can save the updated control file as codeml-M0_branch.ctl and execute CODEML from the command line:

codeml codeml-M0_branch.ctl

The output has the same format as before, with the only difference being an extra block in the output file with the estimated *d*_N_*/d*_S_ ratios for each branch of the input tree. For instance, the output file of the analysis assuming the chicken lineage as the foreground branch includes the following:

**Figure:**
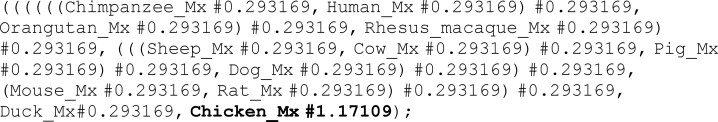


In this tree, the estimated *ω* ratio for every branch is shown as the branch label, after the “#” symbol. Here, all the background lineages have *ω*_0_ = 0.293, while the foreground lineage has *ω*_1_ = 1.171.

In the analysis with the duck lineage labeled as the foreground branch (table 5, test 2), the estimates are *ω*_1_ = 999 for the foreground branch and *ω*_0_ = 0.300 for the background branches. The value 999 is the upper limit set in the program and means infinity. Such extreme estimates can occur if there is a lack of synonymous substitutions along the concerned branch (Hou et al. 2007). Note that, in such cases, the LRT is still valid even though it is hard to estimate the precise value of *ω*_1_. When the chicken and the duck lineages are both labeled as the foreground branches (test 3), the estimates are *ω*_0_ = 0.287 and *ω*_1_ = 0.711, the same estimates we get for test 4 when all three branches in the bird clade are labeled as foreground branches. The results suggest that the chicken and duck lineages have higher *ω* ratios than the other lineages, indicating possible positive selection.

To test the significance of our hypotheses, we can use the LRT (table 5). According to the LRT, we conclude that the branch model better fits the data than the **M0** model for all the hypotheses tested. In other words, the *ω* ratios for the lineages tested under each hypothesis (chicken, duck, or the bird clade) are significantly different from the *ω* ratios for the background branches.

Here, we have shown (1) how to execute CODEML under the branch model when assuming different selective pressures (i.e., different *ω* ratios) for different evolutionary linages or branches on the tree and (2) how to conduct an LRT for positive selection affecting prespecified branches using **M0** as the null model. We have conducted four tests, partly to illustrate that a rooted tree may be used if the model specifies different evolutionary processes for the two branches around the root. Note that the identification of the foreground branches in the test depends on the biological hypothesis being tested, which should be identified *a priori*. If one conducts the branch test with each branch on the tree in turn designated as the foreground without any *a priori* hypothesis, a correction for multiple testing will be necessary.

### Branch-site Model With Heterogeneous *ω* Across Branches and Sites

In this section, we run CODEML under *branch-site* models, in which *ω* is assumed to vary both among lineages and across sites (fig. 3*D*). Such models may be used to detect positive selection affecting specific amino acid sites along prespecified *foreground* branches.

The tree file will have the same format as that the branch models, with the foreground branches tagged with #1. We use the tree files created earlier for the branch models and edit the control files for the branch models as follows:

outfile = out_chicken_branchsite.txt  * Path to the output file

model   = 2 * Enable 2 or more w for branches

NSsites = 2 * Run under model M2a

We can save this file as codeml-branchsite.ctl and execute CODEML from the command line:

codeml codeml-branchsite.ctl

The output includes a section listing the estimate of the proportions for the *k* = 4 site classes assumed in the branch-site model A and the *ω* values for the background and foreground branches (table 6). Site class **0** varies from 0 to 1 (0 < *ω*_0_ < 1), and sites in this class are under purifying selection for both background and foreground branches. In site class **1**, *ω* is always fixed to *ω*_1_ = 1. Those sites are under neutral evolution. In site classes **2a** or **2b**, the foreground branches are under positive selection with *ω*_2_ ≥ 1, while the background branches are under purifying selection with 0 < *ω*_0_ < 1 (site class **2a**) or undergoing neutral evolution with *ω*_1_ = 1 (site class **2b**).

**Table 6.** Maximum-likelihood Estimates of Parameters in the *ω* Distribution Under the Branch-site Model A.

Foreground branch	Site class	Proportion	Background *ω*	Foreground *ω*
Chicken	0	*p*_0_ = 0.387	*ω*_0_ = 0.074	*ω*_0_ = 0.074
	1	*p*_1_ = 0.289	*ω*_1_ = 1	*ω*_1_ = 1
	2a	*p*_2*a*_ = 0.186	*ω*_0_ = 0.074	*ω*_2_ = 2.381
	2b	*p*_2*b*_ = 0.138	*ω*_1_ = 1	*ω*_2_ = 2.381
Duck	0	*p*_0_ = 0.491	*ω*_0_ = 0.088	*ω*_0_ = 0.088
	1	*p*_1_ = 0.356	*ω*_1_ = 1	*ω*_1_ = 1
	2a	*p*_2*a*_ = 0.088	*ω*_0_ = 0.088	*ω*_2_ = 999
	2b	*p*_2*b*_ = 0.064	*ω*_1_ = 1	*ω*_2_ = 999
Duck and chicken	0	*p*_0_ = 0.430	*ω*_0_ = 0.065	*ω*_0_ = 0.065
	1	*p*_1_ = 0.311	*ω*_1_ = 1	*ω*_1_ = 1
	2a	*p*_2*a*_ = 0.150	*ω*_0_ = 0.065	*ω*_2_ = 2.269
	2b	*p*_2*b*_ = 0.108	*ω*_1_ = 1	*ω*_2_ = 2.269
Bird clade	0	*p*_0_ = 0.413	*ω*_0_ = 0.058	*ω*_0_ = 0.058
	1	*p*_1_ = 0.288	*ω*_1_ = 1	*ω*_1_ = 1
	2a	*p*_2*a*_ = 0.177	*ω*_0_ = 0.059	*ω*_2_ = 1.812
	2b	*p*_2*b*_ = 0.123	*ω*_1_ = 1	*ω*_2_ = 1.812

Note.—999 is the upper limit set by the program for the *ω* ratio, which means *ω* = ∞. The branch-site model assumes four site classes (0, 1, 2a, 2b), with different *ω* ratios for the foreground and background lineages. Sites from site class 0 are under purifying selection along all branches with 0 < *ω*_0_ < 1, while all branches in site class 1 are undergoing neutral evolution with *ω*_1_ = 1. In site classes 2a and b, there is positive selection along foreground branches with *ω*_2_ > 1, while the background branches are under purifying selection with 0 < *ω*_0_ < 1 or undergoing neutral evolution with *ω*_1_ = 1.

The estimates of *ω* (table 6) suggest that there are sites under positive selection along the chicken and duck lineages. Indeed, positive selection appears to affect all three branches in the bird clade (fig. 1).

In a new directory, we make a copy of the control file used above, but change the following variables so that the value of *ω* for the foreground branches in site class 2a (*ω*_2_) is not estimated, but instead fixed (fix_omega = 1) to 1 (omega = 1):

fix_omega = 1 * The value of ω_2_ for foreground branches will be fixed

omega = 1 * The fixed value will be ω_2_ = 1

The program will estimate *ω*_0_ and the proportions for site classes as before. We can save the new control file as codeml-branchsite_null.ctl and execute CODEML:

codeml codeml-branchsite_null.ctl

We collect the log-likelihood scores under the null hypothesis as well as in those under the alternative hypotheses from the analysis above to conduct the LRT. The results are summarized in table 7, which show that the duck and chicken myxovirus genes are under positive selection.

**Table 7.** Branch-site Test of Positive Selection Along the Chicken or Duck Branches.

Model	*ℓ*	Free parameters	df	2Δℓ
Chicken as the foreground (unrooted)				
Null model (model A with *ω*_2_ = 1)	ℓ_0_ = −11,946.43	28	1	4.11
Alternative model (model A)	ℓ_1_ = −11,944.38	29		
Duck as the foreground (unrooted)				
Null model (model A with *ω*_2_ = 1)	ℓ_0_ = −11,960.22	28	1	13.27
Alternative model (model A)	ℓ_1_ = −11,953.59	29		
Duck and chicken as the foreground (unrooted)				
Null model (model A with *ω*_2_ = 1)	ℓ_0_ = −11,934.04	28	1	9.93
Alternative model (Model A)	ℓ_1_ = −11,929.07	29		
Bird clade as the foreground (rooted)				
Null model (model A with *ω*_2_ = 1)	ℓ_0_ = −11,927.41	29	1	6.48
Alternative model (Model A)	ℓ_1_ = −11,924.17	30		

Note.—The critical values are $\chi_{1,5\text{\%}}^{2}=3.84$ (i.e., 1 degree of freedom, df, at 5% significance), $\chi_{1,1\text{\%}}^{2}=6.63$ (i.e., 1 degree of freedom, df, at 1% significance), $\chi_{2,5\text{\%}}^{2}=5.99$ (i.e., 2 degree of freedom at 5% significance), and $\chi_{2,1\text{\%}}^{2}=9.21$ (i.e., 2 degree of freedom at 1% significance). The LRT statistic, 2Δℓ, is reported for all model comparisons.

The results for the BEB analysis are reported for all the hypotheses in the corresponding output files. Table 8 shows the sites in the lineages selected as foreground which have a probability higher than 95% or higher than 99% to be positively selected.

**Table 8.** Sites that Have a Probability Higher Than 95% or 99% to be Under Positive Selection According to the BEB Analysis Under the Branch-site Model A.

Hypothesis tested	Pr(ω>1) > 95%	Pr(ω>1) > 99%
Chicken	40, 117	275
Duck	87, 182, 468, 542	–
Duck and chicken	40, 62, 117, 126, 199, 275, 366, 639, 642	–
Bird clade	40, 62, 117, 126, 199, 256, 275, 355, 366, 399, 628, 639, 642	–

Here, we have shown (1) how to execute CODEML under the branch-site model A (Yang et al. 2000; Zhang et al. 2005) and (2) how to run an LRT for positive selection when using the branch-site model A with *ω*_2_ = 1 as a null model (i.e., one less free parameter). The results for the four tests for positive selection are unequivocal: the myxovirus sequences for both the duck and the chicken lineages (and indeed for all three branches of the bird clade) are under positive selection.

## Analyses of Genome-scale Data Sets

Nowadays, it is common to apply the same CODEML analysis to thousands of protein-coding genes from the same set of species. We consider it essential that the users understand the model assumptions and rationale underlying the tests before running such analyses *en masse*. For instance, it may be helpful to first analyze one gene alignment in detail, as explained in our protocol above.

We have modified CODEML to conduct the same test of positive selection using multiple gene alignments from the same set of species, allowing for the possibility that some genes may be missing from some species. All gene alignments will be in the same input sequence file, one after another, with the number of gene alignments specified using the ndata variable in the control file. A main tree for all species is provided in the tree file, from which the tree for each gene alignment is generated by CODEML by pruning off missing species for the gene.

Specifically, the variable ndata is implemented with four options, briefly described here. We provide illustrative examples in the examples/ndata/folder in the PAML release, and a tutorial with additional examples is included in our GitHub repository (https://github.com/abacus-gene/paml-tutorial/tree/main/positive-selection/02_extra_analyses). The options are the following, all assuming an example sequence file with three alignments:

Case ***a***: ndata = 3

Case ***b***: ndata = 3 separate_trees

Case ***c***: ndata = 3 maintree 1

Case ***d***: ndata = 3 maintree 0

In case ***a***, the sequence file has three alignments, all of which are analyzed using the same tree (i.e., one tree block, which consists of the header and the tree in Newick format). This option works only if all gene alignments have all species or sequences present in the tree (i.e., no missing data) and have the same names.

In case ***b***, each alignment has its own block of trees. CODEML will read the first gene alignment in the sequence file and the first block of trees in the tree file to run the analysis. It will then move to the next alignment and the next block of trees and will continue until all alignments are analyzed. In this case, some species may be missing for some genes and the species or sequence names may differ among alignments. The output information for the different gene alignments and different trees are separated by headings like “Dataset 1” and “TREE #1”, respectively.

In case ***c***, the tree file contains only one main tree, which includes all species present in the sequence file. The program will read the gene alignments one by one and generate the subtree corresponding to each gene alignment by pruning off the missing species from the main tree. Then, it will run the ML analysis of each gene alignment and estimate the model parameters and calculate the log-likelihood values.

Case ***d*** is the same as case ***c*** except that CODEML will not conduct the ML analysis. Instead, CODEML will write those gene trees in a text file called genetrees.trees. Compared with case ***c***, case ***d*** is a dry run, useful for checking for errors in the sequence file.

If all species are present in each gene alignment in the sequence file, all four cases should be equivalent, and case ***a*** will be the simplest.

When ndata is used to analyze many gene alignments under the *branch* and *branch-site* models but some species are missing for particular genes, the user should exercise caution. Under these models, branches on the main tree are classified into *foreground* and *background* branches using tags. However, pruning off missing species from the main tree for a particular gene may cause two or more branches on the main tree to be merged into one branch. If those merged branches have the same tag, CODEML will retain the tag for the resulting branch. However, ambiguities will arise when the tags for those branches are different, in which case, CODEML will abort with an error message. Examples about these issues are further discussed in our GitHub repository (https://github.com/abacus-gene/paml-tutorial/tree/main/positive-selection/02_extra_analyses).

## Discussion

### Phylogenetic Methods for Detecting Positive Selection

In this protocol, we have focused on four types of codon-substitution models implemented in CODEML useful for testing positive selection: (1) the one-ratio model with one *ω* for all sites and branches, (2) *site* models with heterogenous *ω* across sites in the coding sequence, (3) *branch* models with different *ω* ratios among branches of the phylogeny, and (4) *branch-site* models with heterogenous *ω* across both branches and sites.

Here, we provide a brief overview of alternative phylogenetic software tools for detecting positive selection using protein-coding genes (see table 9 for a selection of these tools). Note that our protocol does not cover population genetics methods for detecting selective sweeps, which are suited to genomic data sampled from multiple individuals of the same species (Nielsen 2005).

**Table 9.** A Selection of Available Tools for Phylogenetic Tests of Positive Selection.

Tool	Description	Online tutorials	Models implemented to test for positive selection	Language required?	OpenMP/MPI?	Same models as in CODEML?	Citations
**CODEML**	Software that can be used to detect positively selected protein-coding genes and infer parameters of codon-substitution models by fitting SM, BM, and BSM using ML	GitHub: https://github.com/abacus-gene/paml-tutorial	SM: M0, M1a, M2a, M7, M8, M8a BM: M0, two-ratio model, free-ratio model BSM: A, A-null	Bash scripting	No	–	Yang (2007)
**EasyCodeML**	Visual software that implements some **CODEML** models for detecting selection in molecular evolutionary analysis	GitHub: https://github.com/BioEasy/EasyCodeML	SM: M0, M1a, M2a, M7, M8 BM: M0, two-ratio model, free-ratio model BSM: A, A-null	No, it is a Java-based GUI	NA	Yes	Gao et al. (2019)
**ete-evol**	Software that automates the use of preconfigured evolutionary models that are part of **CODEML** and **SLR**	http://etetoolkit.org/documentation/ete-evol/	SM: M0, M1a, M2a, M7, M8 BM: M0, two-ratio model, free-ratio model BSM: A, A-null	Bash scripting	Yes	Yes	Huerta-Cepas et al. (2016)
**FastCodeML**	Software that optimizes positive selection inference by parallelizing the BSM in **CODEML**	No	BSM: A	Bash scripting	Yes	Yes, but optimized to be parallelized	Valle et al. (2014)
**HyPhy/Datamonkey**	Software for comparative sequence analysis with stochastic evolutionary models	http://hyphy.org/	SM: SLAC, FEL, MEME BSM: aBSREL	HyPhy Batch Language (HBL) + Bash scripting	Yes	No	Kosakovsky Pond et al. (2005)
**SLR**	Software that detects sites in the alignment that are unusually conserved and/or unusually variable	No	SM: SLR	Bash scripting	No	No	Massingham and Goldman (2005)

Note.—BM, branch models; BSM, branch-site model; SM, site models.

A number of authors have developed models to account for variable selective pressures among amino acid sites in the protein, similarly to the site models in CODEML. An ML method assigns and estimates a free *ω* ratio for every codon site in the gene, and applies an LRT of the null hypothesis *ω* = 1 using data at one site. This approach is followed by the sitewise likelihood ratio method of Massingham and Goldman (2005) in the SLR program or the fixed-effects likelihood (FEL) method in HyPhy (Kosakovsky Pond et al. 2005); see also Suzuki (2004). A difficulty with this procedure, however, is its use of many parameters in the model (with one *ω* parameter for every site), but it may be effective if a large number of sequences are available in the alignment.

The program HyPhy implements several models that are similar to the site and branch-site models in Codeml. The site models implemented in HyPhy (Kosakovsky Pond et al. 2005; see also Mayrose et al. 2007) use three site classes for synonymous rates as well as three site classes for nonsynonymous rates, which allows the synonymous rate to vary among sites, a feature which some argue is important (Wisotsky et al. 2020). Posterior probabilities that each site belongs to those site classes are calculated using the NEB approach of Nielsen and Yang (1998), allowing positive-selection sites with *ω* > 1 to be identified. The mixed effects model of evolution (**MEME**) (Murrell et al. 2012) and the adaptive branch-site random effects (**aBSREL**) model (Smith et al. 2015) in HyPhy allow the *ω* ratio to vary both among lineages and among sites, similar to the branch-site model discussed in this protocol. However, these models do not require the foreground branches to be specified *a priori*, and instead search for lineages along which some sites appear to be under positive selection with *ω* > 1. This approach may be effective for exploratory data analysis, but caution should be exercised in the interpretation of the LRT (Murrell et al. 2012). Note that in standard hypothesis testing, the null hypothesis should be formulated *a priori* and should not be derived from the data, which are in turn used to test the hypothesis.

A more efficient implementation of the branch-site model A is available in FastCodeML (Valle et al. 2014). The parallel version may be 100 times more efficient than CODEML, making it feasible to analyze large phylogenomic data sets. Several online tools and resources with friendly graphical user interfaces (GUIs) have integrated CODEML as a tool for detecting positive selection. For example, EasyCodeML (Gao et al. 2019) is a wrapper for CODEML, so that the user does not have to run CODEML at the command line. Similarly, ete-evol uses preconfigured evolutionary models in CODEML and SLR to automate the analyses in a parallel manner, as well as an in-built LRT and facilities for plotting the results (Huerta-Cepas et al. 2016).

### Assumptions, Limitations, and Perspectives

The branch models, site models, and branch-site models discussed in this protocol are among the simplest models of codon substitution. They are commonly used in analysis of genomic sequence data to detect genes or sites potentially under positive selection driving adaptation at the molecular level. Our protocol is aimed at beginners, and we do not attempt to provide a comprehensive coverage of the topic. The interested reader may refer to many reviews that discuss the theory, limitations, and applications of those models, such as Yang and Bielawski (2000), Nielsen (2001), Anisimova and Kosiol (2009), Chapter 12 of Yang (2014), and the edited book by Cannarozzi and Schneider (2012), as well as the CODEML program manual and our GitHub repository with the code and data needed to reproduce the results described in this protocol.

Like any statistical test of hypothesis, tests of positive selection discussed here may suffer from two types of errors: the false negative error (or lack of power) and the false positive error. A few simulations have been conducted to examine both types of errors for tests based on the site models and the branch-site models discussed in this protocol (e.g., Anisimova and Yang 2007). Note that all positive selection tests consider excessive nonsynonymous substitutions—beyond the expectation based on the genetic code table and on features of the mutation process at the DNA level—as the evidence for positive selection acting on a given protein. Given the constraints on any functional protein, episodic positive selection may not elevate the nonsynonymous rate high enough for the signal to be detected, in which case the test will have low power. When a test under the site or the branch-site model returns a nonsignificant result, the lack of power of the test could always explain such results. Similarly, when the assumptions of the underlying models are seriously violated, the test may produce excessive false positives (Anisimova and Yang 2007).

First, all models assume that the alignment is correct. Note that excessive alignment errors have been noted to cause false positives for tests based on the site models in simulations (Fletcher and Yang 2010). In particular, some alignment methods tend to lump nonhomologous sites into one column, creating apparent nonsynonymous substitutions at the site, and so it may be prudent to remove regions that are difficult to align before inferring the gene alignment. Alternatively, an approach to align the sequences and fit the codon models jointly using a model of insertions and deletions as well as codon-substitution models could be used, as in the program BAli-Phy (Redelings 2021). Unfortunately, this may not be computationally feasible for large data sets. Interestingly, regions of the proteins undergoing adaptive evolution with many nonsynonymous substitutions (e.g., the hypervariable region of the HIV *env* gene) are noted to also have many insertions and deletions, suggesting that insertion and deletion mutations may also be under positive selection.

Second, commonly used codon-substitution models assume simple mutations or substitutions that change only one nucleotide at a time. Complex substitutions that simultaneously change two or more nucleotides have been noted to cause excessive false positives (Kosiol et al. 2007; Jones et al. 2018; Venkat et al. 2018).

Third, site-based tests are shown to be robust to low levels of recombination among sites of the same gene (Anisimova et al. 2003), although the false positive rate can be very high at high recombination rate.

Finally, biased gene conversion may also cause false positives (Galtier and Duret 2007; Ratnakumar et al. 2010). Several efforts have been made to try and filter out data affected by alignment errors, recombination, or gene conversion before conducting tests of positive selection. Besides simulation analyses, tests of positive selection have also been applied to well-known genes undergoing accelerated evolution such as the HLA (Hughes and Nei 1990). In some cases, the biological hypotheses generated from the statistical test prompted experimental verification, providing exciting case studies of molecular adaption (e.g., Sawyer et al. 2005).

Since the introduction of models of codon substitution about two decades ago (Goldman and Yang 1994; Muse and Gaut 1994), they have been under active development and extended in many ways. These models provide an important framework for studying the direction and strength of natural selection on gene sequence evolution and for detecting molecular adaptation. They also provide more realistic and interpretive models of amino acid replacements, which can be used to infer phylogenetic trees or reconstruct ancestral protein sequences (Yang et al. 1998). More recent efforts have made use of the mutation-selection (Mut-Sel) formulation to improve the biological realism and the interpretive power of the models (Halpern and Bruno 1998; Yang and Nielsen 2008). In particular, Mut-Sel codon models have been used to estimate the distribution of selection coefficients at individual amino acid sites by using large phylogenetic data sets (Rodrigue et al. 2010; Tamuri et al. 2014; Tamuri and dos Reis 2022), to estimate the mutation bias (Latrille and Lartillot 2022), and to test for association between the rate of gene sequence evolution and life-history traits of the species (Latrille et al. 2021). By explicitly considering mutational biases, fixation probabilities of synonymous and nonsynonymous mutations, Mut-Sel models have greater interpretive power and have the potential of integrating population genetics and phylogenetics to further our understanding of the evolutionary process of protein-coding genes.

## Supplementary Material

msad041_Supplementary_Data

## Data Availability

The code and data needed to reproduce the results described in this protocol can be found in the GitHub repository positive-selection available at https://github.com/abacus-gene/paml-tutorial. In brief, the repository includes a step-by-step tutorial for downloading the sequences used in Hou et al. (2007), generating the alignment, reconstruct the phylogeny, and conducting the analyses as described in the protocol. All in-house scripts and software used are also provided in the repository. In addition, we provide an example dataset and instructions for running the newly implemented options for analyzing multiple gene alignments described in the text.
