# Supplementary material for: Beginner's Guide on the Use of PAML to Detect Positive Selection
Source: Mol Biol Evol. 2023 Apr 25;40(4):msad041. doi: 10.1093/molbev/msad041 (PMC10127084; doi:10.1093/molbev/msad041)
Supplement: msad041_Supplementary_Data [file msad041_supplementary_data.pdf]

SUPPLEMENTARY MATERIAL

Beginner's guide on the use of PAML to detect positive selection

Sandra Álvarez-Carretero [1,#], Paschalia Kapli [1,#], and Ziheng Yang [1*]

[1] Department of Genetics, Evolution and Environment, University College London, London, United Kingdom

*Corresponding author: z.yang@ucl.ac.uk.

# These authors contributed equally.

Alignment, sequence data file, and tree file

Before positive selection inference can take place, users are responsible for ensuring that the molecular alignment and corresponding phylogeny have been properly estimated.

While the scope of this protocol is not focused on guiding users for alignment or molecular phylogeny inference, we outline below some checks that we encourage users to carry out before running `CODEML`:

1. Guaranteeing that the correct sequence data have been downloaded is extremely important. Users need to make sure that the downloaded data are not contaminated or mislabeled before generating the molecular alignment (i.e., the sequence really corresponds to the correct gene and species).
2. A number of alignment programs can be used to generate the codon alignment to be used with `CODEML`, including `PRANK` (Löytynoja and Goldman 2005, 2008) and `PAGAN` (Löytynoja et al. 2012). Use the option for aligning coding sequences if such an option exists. To avoid out-of-frame indels, one strategy for aligning coding nucleotide sequences is to align the translated protein sequences first and then use protein alignment to generate the codon alignment. A number of online tools can be used for this purpose, including `PAL2NAL` (Suyama et al. 2006) and `TranslatorX` (Abascal et al. 2010) (see our GitHub repository (https://github.com/abacus-gene/paml-tutorial/tree/main/positive-selection/00_data) for more details).
3. Often the alignments generated by the alignment programs are in the FASTA format instead of the PHYLIP format, as required by `CODEML`. In the GitHub repository, we include a PERL script (`FASTAtoPHYL.pl` (https://github.com/abacus-gene/paml-tutorial/blob/main/positive-selection/00_data/scripts/FASTAtoPHYL.pl)) for the conversion.
4. We recommend removing regions of the alignment that are predominantly gaps or are otherwise hard to align. Some software such as `GUIDANCE` (Penn et al. 2010) may be

useful to assist the user to delete or mask unreliable alignment regions. Stop codons must be removed.

5. The aligned sequences may be analyzed using a phylogeny-reconstruction program to infer the phylogenetic tree for the gene. For example, `RAxML-NG` (Kozlov et al. 2019), the successor of `RAxML` v8.2.10 (Stamatakis 2014), can be used to infer the maximum-likelihood tree under a variety of nucleotide-substitution models. Branch lengths in the generated tree should be removed as they may interfere with the tags for labelling branches used by `CODEML`. We include some code snippets in [the step-by-step tutorial in the GitHub repository](https://github.com/abacus-gene/paml-tutorial/tree/main/positive-selection/00_data#readme) (https://github.com/abacus-gene/paml-tutorial/tree/main/positive-selection/00_data#readme) that can help users include said tags.

Gene tree versus species tree

Sometimes, the gene tree (e.g., the ML tree inferred using the gene alignment) and the well-established species tree may differ. Should analysis of positive selection be based on the gene tree or species tree? This question does not have a simple answer. Note that the models used in the test assume that the phylogenetic tree represents the true evolutionary relationships of the sequences. Consequently, one should use whichever tree is most likely to be correct. In analysis of duplicated genes with orthologs and paralogs, the species tree may not be applicable so that the inferred gene tree is the only choice. Similarly, in analysis of viral sequences, a species tree does not exist, and hence the gene tree is the only choice. If the gene sequences are short or otherwise do not contain much phylogenetic information, the inferred gene tree may be unresolved or incorrect, and the species tree will be preferable. If convergent evolution is likely to have misled gene tree reconstruction, the species tree will be preferable.

When the phylogenetic tree is in doubt, it is advisable to assess the impact of the tree topology by using several plausible trees (e.g., including the ML tree for the gene and the species tree). In simulation analyses, site-based tests of positive selection are found to be robust to minor changes to the phylogeny (e.g., Yang et al. 2000). In branch or branch-site tests, if the foreground branches are well-resolved lineages and the phylogenetic uncertainties concern details inside a clade designated the background branches, the tree topology may not be expected to have a major impact on the test. For additional checks to ensure the quality of inferences of positive selection, see Álvarez-Carretero and dos Reis 2020).

Rooted versus unrooted trees

In PAML, rooted trees are represented using a trifurcation at the root while unrooted trees are binary at the root. For example, in Figure S1A, the left tree "`(A, B), C));`" is a rooted tree with four branch lengths including two branch lengths around the root, while the right tree "`(A, B,`

C);" is an unrooted tree with three branches (the branch lengths around the root, b_{3a} and b_{3b}, are merged into one branch length, b_3). You can use a text editor or various scripts to remove a pair of parentheses in the Newick notation to convert a rooted tree into an unrooted one. We include a code snippet in our GitHub tutorial (https://github.com/abacus-gene/paml-tutorial/tree/main/positive-selection/00_data#readme).

Whether rooted or unrooted trees should be used in the analysis depends on whether the substitution model can identify the root of the tree. In particular, if the substitution model is time-reversible, the substitution process is time-homogeneous: the nucleotide, codon, or amino acid frequencies are stationary and different lineages have their own rates (i.e., without the assumption of the molecular clock). In this case, the location of the root is unidentifiable and unrooted trees should be used. Virtually, all phylogenetic programs such as `RAxML` or `IQ-TREE` (Minh et al. 2020) assume time-reversible substitution models and no clock. And hence generate unrooted trees. Even if the tree-drawing software (e.g., `FigTree`) may display a rooted tree for visual purposes, the tree should be considered unrooted if it is inferred under a model that cannot identify the root.

Almost all codon models developed in the literature, including those discussed here, are time-reversible models. In addition, we do not assume the molecular clock in the analyses described throughout the protocol. As a result, an unrooted tree should in general be used with one exception when using branch or branch-site models (see the protocol for an example). In this case, if we assume that the two branches around the root are undergoing different evolutionary process (e.g., with different ω), the location of the root is identifiable, and a rooted tree should be used. If the two branches around the root are assumed to evolve according to the same process (e.g., both branches are foreground branches or both branches are background branches), the root is unidentifiable, and an unrooted tree should be used. Under model **`M0`** (one-ratio) and the sites models (e.g., **`M1a`**, **`M2a`**, **`M7`**, **`M8`**), the two branches around the root are always assumed to evolve according to the same process, and hence an unrooted tree should be used. Several scenarios are illustrated in Figure S1, in which black branches represent foreground branches and gray branches are background branches.

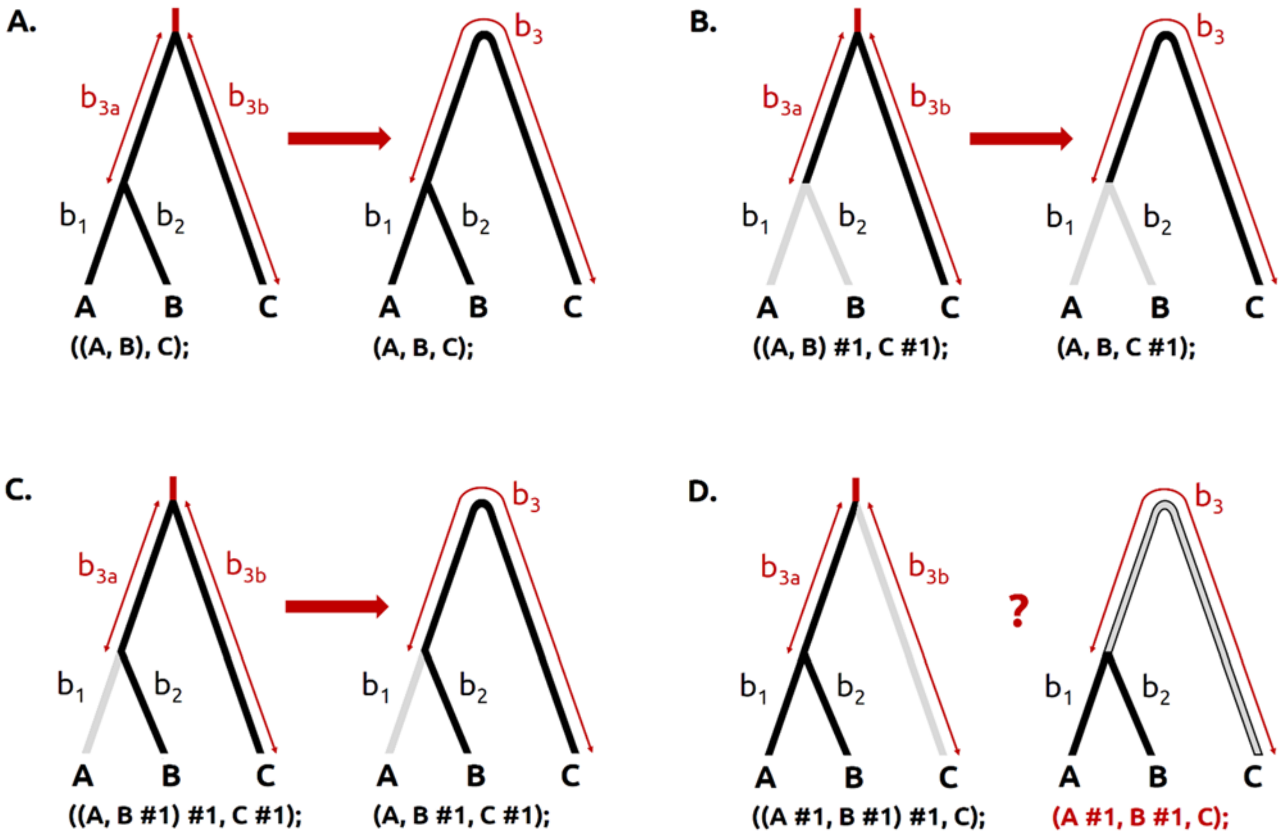

Figure S1. Rooted and unrooted trees for fitting codon models. Black branches are foreground branches while gray branches are background branches. In A, B, and C, the two branches around the root are assumed to have the same evolutionary process and unrooted trees should be used. In D, the two branches around the root have different evolutionary process (one branch is foreground and the other is background), and the rooted tree on the left should be used. Use of the unrooted tree on the right would specify a different model.

Before running any analyses, users may ask: what is the impact of incorrectly using a rooted tree when the unrooted tree should be used instead? To answer this query, let's consider fitting model **`M0`** (one-ratio) to the rooted tree on the left in Figure S1A. All model parameters such as the transition/transversion rate ratio (κ), the nonsynonymous/synonymous rate ratio (ω), and the branch lengths b_1 and b_2 will be identifiable and correctly estimated, and the log likelihood value will be correctly calculated. The branch lengths b_{3a} and b_{3b} , however, are not estimable although their sum $b_3 = b_{3a} + b_{3b}$ is. If one runs `CODEML` multiple times, the estimates of b_{3a} and b_{3b} may vary among runs, but the estimate of b_3 will be stable. If we conduct the LRT of the null hypothesis ($\omega = 1$) and use the rooted tree in both the null and alternative hypotheses, we will be overcounting the number of parameters by 1 (i.e., the additional branch length used to root the tree), but the degree of freedom will be correctly calculated, and the LRT will still be correct. For instance, if the rooted tree is used under both **`M1a`** and **`M2a`**, the LRT will be correct even if the number of parameters is over-counted by one. Note that this scenario also applies to the site

tests. In those cases, ideally the unrooted tree should be used, although using the rooted tree does not incur any serious harm as previously explained.

If we now consider the branch or branch-site models specified in Figure S1D left, the two branches around the root are assumed to have different evolutionary processes. This model can be only expressed by using the rooted tree as using the unrooted tree would specify a completely different model.

BEB Analysis

In the example used in the protocol, the BEB analysis did not list any site as positively selected with a probability larger than 95% or larger than 99%. Below, we show an example of how the output would look like had a site been positively selected under the restrictions mentioned above:

```
Bayes Empirical Bayes (BEB) analysis (Yang, Wong & Nielsen 2005. Mol. Biol.
Evol. 22:1107-1118)
Positively selected sites (*: P>95%; **: P>99%)
(amino acids refer to 1st sequence: Rhesus_macaque_Mx)

            Pr(w>1)     post mean +- SE for w

    10 S      0.984*          1.468 +- 0.634
    25 S      0.999**         1.464 +- 0.638
```

Under this fictitious scenario, the 10th site in the alignment has a posterior probability 98.4% of coming from the positive-selection class with $\omega > 1$. The approximate posterior distribution of ω for the site has mean 1.468 and SD 0.634. Similarly, site 25 has a posterior probability 99.9% of coming from the positive-selection class, with approximate posterior mean for ω to be 1.464 and SD 0.638. In the `CODEML` output, posterior probabilities $P > 0.95$ are indicated by * and those with $P > 0.99$ are indicated by **.

References

Abascal F., Zardoya R., Telford MJ.T. 2010. TranslatorX: multiple alignment of nucleotide sequences guided by amino acid translations. Nucleic Acids Res. 38(Web Ser:W7-13.

Álvarez-Carretero S., dos Reis M. 2020. Bayesian phylogenomic dating. In: Ho S.Y.W., editor. The Molecular Evolutionary Clock. Springer.

Kozlov A., Darriba D., Flouri T., Morel B., Stamatakis A. 2019. RAxML-NG: a fast, scalable and user-friendly tool for maximum likelihood phylogenetic inference. Bioinformatics. 35:4453–4455.

Löytynoja A., Goldman N. 2005. An algorithm for progressive multiple alignment of sequences with insertions. Proc. Natl. Acad. Sci. U.S.A. 102:10557–10562.

Löytynoja A., Goldman N. 2008. Phylogeny-aware gap placement prevents errors in sequence alignment and evolutionary analysis. Science. 320:1632–1635.

Löytynoja A., Vilella A., Goldman N. 2012. Accurate extension of multiple sequence alignments using a phylogeny-aware graph algorithm. Bioinformatics. 28:1684–1691.
Minh B., Schmidt H., Chernomor O., Schrempf D., Woodhams M., von Haeseler A., Lanfear R. 2020. IQ-TREE 2: New models and efficient methods for phylogenetic inference in the genomic era. Mol Biol Evol. 37:1530–1534.
Penn O., Privman E., Landan G., Graur D., Pupko T. 2010. An alignment confidence score capturing robustness to guide tree uncertainty. Mol Biol Evol. 27:1759–1767.
Stamatakis A. 2014. RAxML version 8: a tool for phylogenetic analysis and post-analysis of large phylogenies. Bioinformatics. 30:1312–1313.
Suyama M., Torrents D., Bork P. 2006. PAL2NAL: robust conversion of protein sequence alignments into the corresponding codon alignments. Nucleic Acids Res. 34:W609–W612.
Yang Z., Nielsen R., Goldman N., Pedersen A.M.K. 2000. Codon-substitution models for heterogeneous selection pressure at amino acid sites. Genetics. 155:431–449.
